# Female tilapia, *Oreochromis* sp. mobilised energy differently for growth and reproduction according to living environment

**DOI:** 10.1038/s41598-024-52864-0

**Published:** 2024-02-05

**Authors:** Ros Suhaida Razali, Sharifah Rahmah, Yu Ling Shirly-Lim, Mazlan Abd Ghaffar, Suhairi Mazelan, Mohamad Jalilah, Leong-Seng Lim, Yu Mei Chang, Li Qun Liang, Young-Mao Chen, Hon Jung Liew

**Affiliations:** 1 Higher Institution Centre of Excellence, Institute of Tropical Aquaculture and Fisheries, Universiti Malaysia Terengganu, 21030 Kuala Nerus, Terengganu Malaysia; 2 Faculty of Fisheries and Food Science, Universiti Malaysia Terengganu, 21030 Kuala Nerus, Terengganu Malaysia; 3 Faculty of Science and Environment Marine, Universiti Malaysia Terengganu, 21030 Kuala Nerus, Terengganu Malaysia; 4 Institute of Marine Biotechnology, Universiti Malaysia Terengganu, 21030 Kuala Nerus, Terengganu Malaysia; 5 Borneo Marine Research Institute, Universiti Malaysia Sabah, Jalan UMS, 88400 Kota Kinabalu, Sabah Malaysia; 6 Heilongjiang River Fisheries Research Institute, Chinese Academy of Fishery Sciences, Harbin, China; 7 Bachelor Degree Program in Marine Biotechnology, College of Life Sciences, National Taiwan Ocean University, Keelung, Taiwan; 8 Center of Excellence for the Oceans, National Taiwan Ocean University, Keelung, Taiwan

**Keywords:** Developmental biology, Ecology, Physiology, Zoology, Climate sciences, Environmental sciences

## Abstract

This study was conducted to investigate the energy mobilisation preference and ionoregulation pattern of female tilapia, *Oreochromis* sp. living in different environments. Three different treatments of tilapia as physiology compromising model were compared; tilapia cultured in recirculating aquaculture system (RAS as Treatment I—RAS), tilapia cultured in open water cage (Treatment II—Cage) and tilapia transferred from cage and cultured in RAS (Treatment III—Compensation). Results revealed that tilapia from Treatment I and III mobilised lipid to support gonadogenesis, whilst Treatment II tilapia mobilised glycogen as primary energy for daily exercise activity and reserved protein for growth. The gills and kidney Na^+^/K^+^ ATPase (NKA) activities remained relatively stable to maintain homeostasis with a stable Na^+^ and K^+^ levels. As a remark, this study revealed that tilapia strategized their energy mobilisation preference in accessing glycogen as an easy energy to support exercise metabolism and protein somatogenesis in cage culture condition, while tilapia cultured in RAS mobilised lipid for gonadagenesis purposes.

## Introduction

Tilapia is one of the most popular household choices because of its affordable price and boneless meat that is perfectly served with a variety of home-cooked recipes. Nevertheless, due the Covid-19 pandemic with restriction in operation under Movement Control Order (MCO) in many countries have limited domestic tilapia supply and whole value chain^1^. MCO not only restricted food supply, but also limited household income. Therefore, many households have started tilapia culture using backyard recirculating aquaculture system (RAS) facility to support family needs and contribute to household income during MCO. However, the growth performance of tilapia in RAS was not comparable to the tilapia from cage culture system^2^. This issue was experienced by most of the backyard household tilapia farmers.

As a mouthbrooder species, tilapia reached sexual maturation and reproduce at the body weight about 100 g^3^. Once the spawning process takes place, female tilapia immediately collect fertilized eggs and incubate in her mouth till the larvae reach free swimming stage^4^. With that, our first hypothesis speculated that tilapia cultured in RAS tend to spend energy for secondary maturation and reproduction, where slow growth is expected. Whereas, we assume that cage-cultured tilapia did not reproduce as the eggs can be fall through the mesh, which has been documented in the earlier study by Pagan-Font (1975)^2^. However, it remains to be tested whether fish continue to reproduce eggs under cage-cultured condition or whether the better growth is achieved due to the fact that the females does not have to incubate the eggs, during which time the females would normally not feed during incubation period. In addition, maintaining homeostasis balance is essential to fulfil basal metabolism needs. Our second hypothesis assume that tilapia cultured in the open cage system would have to maintain a high level of ionoregulatory activity such as NKA activity to maintain a balance homeostasis. Where the ionic composition for cage-cultured water might be different accordingly locality. Thereby, maintaining active NKA activity is needed to facilitate ammonia excretion while retaining internal Na^+^ level^5^, especially when fish actively exercise extra energy expenses to support exercise metabolism^6,7^.

Performing active ionoregulation is an energetically expensive process that requires about 1–20% of the total ATP demand^8,9^. High NKA activity is also expected to associate with ammonia excretion, which also require additional ATP expenditure^10^. Increase ammonia excretion efficiency concurrently induces an increase in NKA activity have also been reported previously in other species such as common carp *Cyprinus carpio*^6,7^, rainbow trout *Oncorhynchus mykiss*^11^ and climbing perch *Anabas testudineus*^10^. With these hypotheses as background, the objective of this study was aimed to investigate the pattern of reserve energy mobilization and ionoregulation of female tilapia cultured in RAS and cage conditions. Thereby, this study was pursued on the tilapia cultured in recirculating aquaculture system (Treatment I—RAS) in comparison to the tilapia cultured from open water cage culture system at Como River, Kenyir Lake (Treatment II—Cage). Additionally, a group of tilapia that were transferred from Treatment II—Cage and cultured in RAS for 4 weeks (Treatment III—Compensation) were used to compare their energy mobilisation and ionoregulation patterns with Treatment I and II tilapia.

## Materials and methods

### Experimental animal ethical approval

Fish handling practice during experimentation was followed and approved by Universiti Malaysia Terengganu Animal Care Committee according to the guidelines of the Laboratory Animal Ethic Regulation (UMT/JKEPHT/2019/38). The ARRIVE rules and guidelines were taken into consideration by the authors.

### Specimen management and experimental design

Hybrid red tilapia used in this study were classed in three different treatments, the experiment system using in this study was referred from common tilapia farming practice either using RAS and cage-cultured in Malaysia. Treatment I—RAS referred to the tilapia cultured in an enclosed recirculating aquaculture system (RAS) at the hatchery facility in Institute of Tropical Aquaculture and Fisheries (AKUATROP), Universiti Malaysia Terengganu (UMT). For Treatment-I, a total of 300 juveniles hybrid red tilapia with an initial average body weight (BW) at 45.25 ± 6.48 g and body length (BL) at 12.89 ± 5.84 cm were purchased from a local farmer who conducted tilapia cage farming at Sungai Como, Tasik Kenyir, Terengganu and distributed equally into the RAS and cultured for 3 months in triplication. Stocking density was set at 50 fish/m^3^ with a total of 100 fish per replicate at volume of 2000 L water with the tank size of 2 × 2 × 1.5 m (L × W × H). This stocking density was set based on the general tank culture practiced by local farmers. Each replicate was equipped with an individual RAS system consisted of external mechano-biofilter with sponges as particle waste separation in the first compartment, fine sponges in the second compartment (Japanese Max Bio-sponges) and bio-balls in the third compartment to promote denitrification. Ceramic balls with a minimum specific surface area of 20,000 ft^2^/ft^3^ were placed in the last compartment before the water flows back into the culture tank. Water in the system was refreshed weekly at 40% and monitored by using the YSI multiple meter (YSI-556 MPS). Water quality was monitored at NH_3_/NH^+^_4_ < 0.25 mg/L, NO^−^_2_ < 0.25 mg/L and NO^−^_3_ < 20 mg/L by using API Freshwater quality test kits (MARS Fishcare, Hamilton, Chalfont, PA, USA). The water exchange is done to ensure the overall health and optimal condition of aquatic organisms by controlling water quality, nutrient levels and other important parameters in the RAS system as regular maintenance to support the growth and well-being of the fish cultured.

For Treatment II—Cage referred to the tilapia cultured in the crystal clear open water floating cages (5°02′22.1″N 102°50′41.1″E) at the Como River, Kenyir Lake, Terengganu. Rectangular cage culture system were built using a combination of wooden frame structure and walkway. Polyethylene barrels were used as floaters at the size of 4 × 4 × 2 m with the net mesh size of 1.5 m^2^. In order to set similar cage sizes with Treatment I—RAS, an additional layer of net was constructed to achieve the size of 2 × 2 × 1.5 m (L × W × H) in triplication. This allow the study to have the same stocking density and culture space for comparison. Therefore, the stocking density was set at 50 fish/m^3^ with the initial BW of 43.82 ± 6.81 g and BL of 12.05 ± 5.64 cm. In-situ physical water parameters both inside and outside the cages were measured using the YSI multiple meter (YSI-556 MPS) and water ion analysis (Ion Chromatographic) as in Table 1.

**Table 1:** Water ions concentration (mOsm/L) from Treatment I—hatchery recirculating aquaculture system (RAS), Treatment II—Como River cage culture (Cage) and Treatment III—RAS (Compensation).

Ions concentration (mOsm/L)	Treatment I (RAS)	Treatment II (Cage)	Treatment III (Compensation)
Na^+^	0.0950 ± 0.0005^a^	0.0776 ± 0.0002^b^	0.0921 ± 0.0014^a^
K^+^	0.0580 ± 0.0002^a^	0.0289 ± 0.0005^b^	0.0523 ± 0.0008^a^
Cl^−^	0.0901 ± 0.0006^a^	0.0547 ± 0.0003^b^	0.0937 ± 0.0003^a^
Ca^2+^	0.1630 ± 0.0008^a^	0.0742 ± 0.0003^b^	0.1618 ± 0.0009^a^
Mg^2+^	0.0611 ± 0.0003^a^	0.0294 ± 0.0003^b^	0.0625 ± 0.0011^a^

The ionic concentrations of sodium (Na^+^), potassium (K^+^), chloride (Cl^−^), calcium (Ca^2+^) and magnesium (Mg^2+^) in water sources from Group I—(RAS) hatchery recirculating aquaculture system tilapia culture system, Group II—(Cage) water sample from Como River cage culture water and Group III—(Compensation) RAS system from compensation. Different superscript letters in the same row indicate significant differences of ionic concentration amongst different types of water (*p* < 0.05). Results are presented as mean ± standard error mean (mean ± SEM).

Treatment III—Compensation referred to the tilapia cultured from Treatment II for two months that were transferred back to the AKUATROP hatchery and cultured in the RAS facility for one month. Fish that were introduced into the RAS system were maintained following the same management practice as mentioned in Treatment I with similar design. Stocking density was set at 50 fish/m^3^ in triplication with a total of 100 fish per tank at a volume of 2000 L. During the cultivation period for all Treatments, water pH was maintained at 7.23 ± 0.59, temperature at 28.5 ± 1.5 °C and dissolved oxygen at 5.8 ± 0.8 mg/L. Throughout the experimental process, feeding was given twice a day at 3% of BW at 8:00 h and 16:00 h using commercial tilapia pellet (28% protein and 3% fat; TP-2 Star-Feed®Star Feedmills (M) Sdn. Bhd., Rawang, Malaysia) for RAS, cage, and compensation treatments, respectively. Feeding rate at 3% per feeding frequency was based on commercial cage-cultured practice.

In order to compare the physiological responses of tilapia living in Treatment I—RAS and Treatment II—Cage conditions, both Treatments of tilapia and water samples were analysed. For Treatment I, a total of 10 female fishes were sampled randomly from each replication. Meanwhile for Treatment II, 10 female fishes were sampled randomly from three different cages after three months culture period. For Treatment III—Compensation, 10 female fishes were sampled randomly from each replication at weekly intervals for four weeks to reveal energy utilization and ionoregulation pattern as compensation strategy of hybrid red tilapia after being transferred from cage culture to RAS culture conditions.

### Sampling procedures

At every sampling intervals, 100 mL water samples from both Treatments were collected for ionic analysis (Table 1). Concurrently, a total of 20 female tilapia were collected randomly for biometric characteristics measurement and tissue collection for biochemistry analysis. During the sampling process, all selected fishes were anesthetized with clove oil at 10 mg/L^12^. The clove oil was first mixed with ethanol to make a stock solution at a ratio of 1:10 (clove oil:ethanol) before use in order to assist emulsification. After fish showed passive operculum movement and loss of equilibrium, they were immediately removed and blotted for biometric measurement followed by blood sampling. Blood was drawn via caudal peduncle using a 1 ml heparinized syringe and carefully expelled into a heparinized 1.5 ml bullet tube. Samples were immediately centrifuged at 5000 g under 4 °C for 30 s. Thereafter, plasma samples were transferred into another 1.5 ml bullet tube and immediately frozen in liquid nitrogen (N_2_). In order to collect other tissues, fish were euthanized with a sharp blow to the head according to the rules of fish welfare. Thereafter, gills, liver, kidney, muscle tissues and gonad (if available) were excised quickly. Wet liver mass was measured and all other tissues were wrapped in aluminium foil individually. All samples were frozen in liquid nitrogen immediately and stored at − 80 °C until analysis. Both liver and muscle tissues were used for bioenergy analysis, while gills and kidney samples were used for enzymatic electrolytes ATPase transporters analysis.

### Biometric measurement

Biometric measurement was used to calculate the condition factor, whereas liver and gonad weights were used to calculate the hepatosomatic index (HSI) and gonadosomatic index (GSI), respectively. Whereas, HSI = 100 × (LW/BW), where LW is the liver weight (g) and BW is the body weight (g) of fish. GSI was calculated as GSI = 100 × (GW/BW), where GW is the ovary weight (g) and BW is the body weight (g) of fish. In this study, GSI was measured only for female tilapia, as the female is the parent performing mouthbrooding incubation with no food intake during this period.

### Tissue metabolites

For bioenergy analysis, 2 g of liver and muscle tissues were homogenized using a handheld homogenizer under ice-chilled condition^13^. Homogenization was performed at 5 × folds dilution factor with ultrapure water (Milli-Q grade). Thereafter, total bioenergy of the liver and muscle tissue were analysed for lipid, protein and glycogen contents. Lipid was extracted by methanol-chloroform and measured with tripalmitin as standard reference^14^. Protein measurement was performed following the Bradford method^15^ using bovine serum albumin as standard reference. Glycogen content was measured using Anthron method with glycogen as standard reference^16^.

### Plasma osmolality and electrolytes

Plasma osmolality levels were measured using Osmometer (Advanced Instrument Inc.—Model 3320) with unit expressed as mOsm/l. Plasma electrolytes such as Na^+^, K^+^, Cl^−^, Ca^2+^ and Mg^2+^ were measured using the Ion Chromatography Analyzer (Metrohm 81 Compact IC Plus—Model 883) with unit expressed as mmol/L.

### Gills and kidney enzymatic Na^+^/K^+^ ATPase activity

Gills and kidney NKA activity was measured according to the method described by^13,17^. A total of 8 samples from each gills and kidney were randomly selected for electrolytes enzymatic ATPase activity analysis. Selected samples were homogenized with the mixture of ice-cooled neutralized SEI/SEID buffer solution (SEI—150 mM sucrose; 10 mM EDTA; 50 mM imidazole solution/SEI with 0.1% sodium deoxycholate solution) with buffer solution pH 7.5 at ratio of 4:1. Thereafter, samples were centrifuged at 5000 g for 1 min at 4 °C to obtain enzyme supernatant. During enzymatic measurement, duplication of 10 µl supernatant samples were pipetted and carefully transferred into 96-wells microplate in two series. A freshly made 200 µl mixture cocktail assay solution A (400 U lactate dehydrogenase; 500 U pyruvate kinase; 2.8 mM phosphoenolpyruvate; 0.7 mM ATP; 0.22 mM NADH; 50 mM imidazole) were added into the first series supernatant and 200 µl mixture cocktail assay solution B (mixture assay A with 0.4 mM ouabain) were added into the second series supernatant on the microplate. The NKA activity was measured kinetically by using a spectrophotometer (MultiskanTM FC microplate photometer, ThermoFisher Scientific™) read at 340 nm for 10 min with 15 s intervals. Adenosine diphosphate (ADP) was used as standard reference. NKA activity was calculated by subtracting the oxidation rate of NADH in the presence of ouabain from the oxidation rate to the NAD in the absence of ouabain. The crude homogenate protein was determined by using bovine serum albumin (US Biochemical, Cleveland, OH, USA) as standard reference and read at 430 nm according to^15^. The NKA activity unit was expressed as μmol ATP/h/mg protein^5^.

### Statistical analysis

The results of growth indication, plasma osmolality, electrolytes, bioenergy and NKA activities were presented as mean ± standard error mean (SEM) (n = 10). Prior to significance analysis, all data were checked for normality distribution by using Shapiro–Wilk test and homogeneity of variance by using Levence test. In case of failure to fulfil normality and homogeneity requirement, data were either log or arcsine square root transformed prior further analysis. Data collected from Treatment I-RAS, Treatment II-Cage and Treatment III-Compensation on weekly progress were compared by using one-way analysis of variance (ANOVA). Tukey HSD post-hoc test was performed to identify significant differences among experimental series treatments set at 95% confident limit at *p* < 0.05.

### Ethics approval and consent to participate

Fish handling practice during experimentation was followed and approved by Universiti Malaysia Terengganu Animal Care Committee according to the guidelines of the Laboratory Animal Ethic Regulation (UMT/JKEPHT/2019/38). The ARRIVE rules and guidelines were taken into consideration by the authors.

## Results

### Biometric indication

In terms of growth performances, no significant differences in BL was noticed (*p* > 0.05), but tilapia cultured in Treatment II had a heavier BW (*p* < 0.05; Table 2). In week-1, highest BW was recorded in Treatment II at 212.11 ± 3.79 g, which was significantly highest compared to Treatment I at 167.69 ± 2.35 g and Treatment III with BW recorded only at 162.31 ± 2.49 g. Whereas, BL was not significantly different for all treatments. As shown in Table 2, the average hepatosomatic index (HSI) for tilapia in Treatment I was recorded at 1.45 ± 0.04, which was significantly higher compared to tilapia from Treatment II at 1.02 ± 0.05 (*p* < 0.05). For Treatment III, the highest HSI was found on week-3 of culture period. The HSI noted at week-1 was 1.23 ± 0.18, had shown a significant increasing pattern to week-2 at 1.51 ± 0.10 and reached the highest at week-3 at 1.95 ± 0.21. However, a slightly decreasing trend was noticed at week-4 with HSI at 1.57 ± 0.11. Whereas, GSI for Treatment I was recorded at 4.48 ± 0.15 and Treatment II at 3.15 ± 0.51, respectively. For Treatment III, GSI at week-1 was recorded at 3.36 ± 0.24, week-2 at 3.45 ± 0.24 and week-3 at 4.09 ± 0.59 were insignificant when compared with Treatment II. However at week-4, GSI was recorded at 4.61 ± 0.56 that was similar with Treatment I and significantly higher compared to Treatment II and those from Treatment III at week-1, -2 and -3, respectively (Table 2).

**Table 2:** Biometric data and growth performances of hybrid red tilapia *Oreochromis* sp. from Treatment I (RAS), Treatment II (Cage) and Treatment III (Compensation).

Biometric	Treatment I	Treatment II	Treatment III			
	RAS	Cage	Compensation			
			Week-1	Week-2	Week-3	Week-4
Final body weight (g)	167.69 ± 2.35^a^	212.11 ± 3.79 ^b^	162.31 ± 2.49^a^	175.59 ± 10.15^ab^	184.92 ± 7.06^ab^	182.44 ± 10.12^ab^
Final body length (cm)	21.12 ± 0.20	20.52 ± 0.31	19.87 ± 0.86	20.26 ± 0.69	20.02 ± 0.37	21.78 ± 0.65
Hepatosomatic index (HSI)	1.45 ± 0.04^b^	1.02 ± 0.05^a^	1.23 ± 0.18^ab^	1.51 ± 0.10^b^	1.95 ± 0.21^c^	1.57 ± 0.11^bc^
Gonadosomatic index (GSI)	4.48 ± 0.15^a^	335 ± 0.51 ^b^	3.36 ± 0.24^b^	3.45 ± 0.24^b^	4.09 ± 0.59^ab^	4.61 ± 0.56^a^

The biometric data of hybrid red tilapia *Oreochromis* sp. from Treatment I (RAS), Treatment II (Cage) and Treatment III (RAS-Compensation) which include final body weight, final body length, HSI and GSI. Different lowercase letters indicate significant differences among Treatments (RAS, Cage and RAS-compensation). Results are presented as mean ± standard error mean (mean ± SEM, *p* < 0.05, n = 10). Since there were no significant differences among the treatments, grouping letters were omitted in Final body length.

### Tissue bioenergy

**Figure 1:**
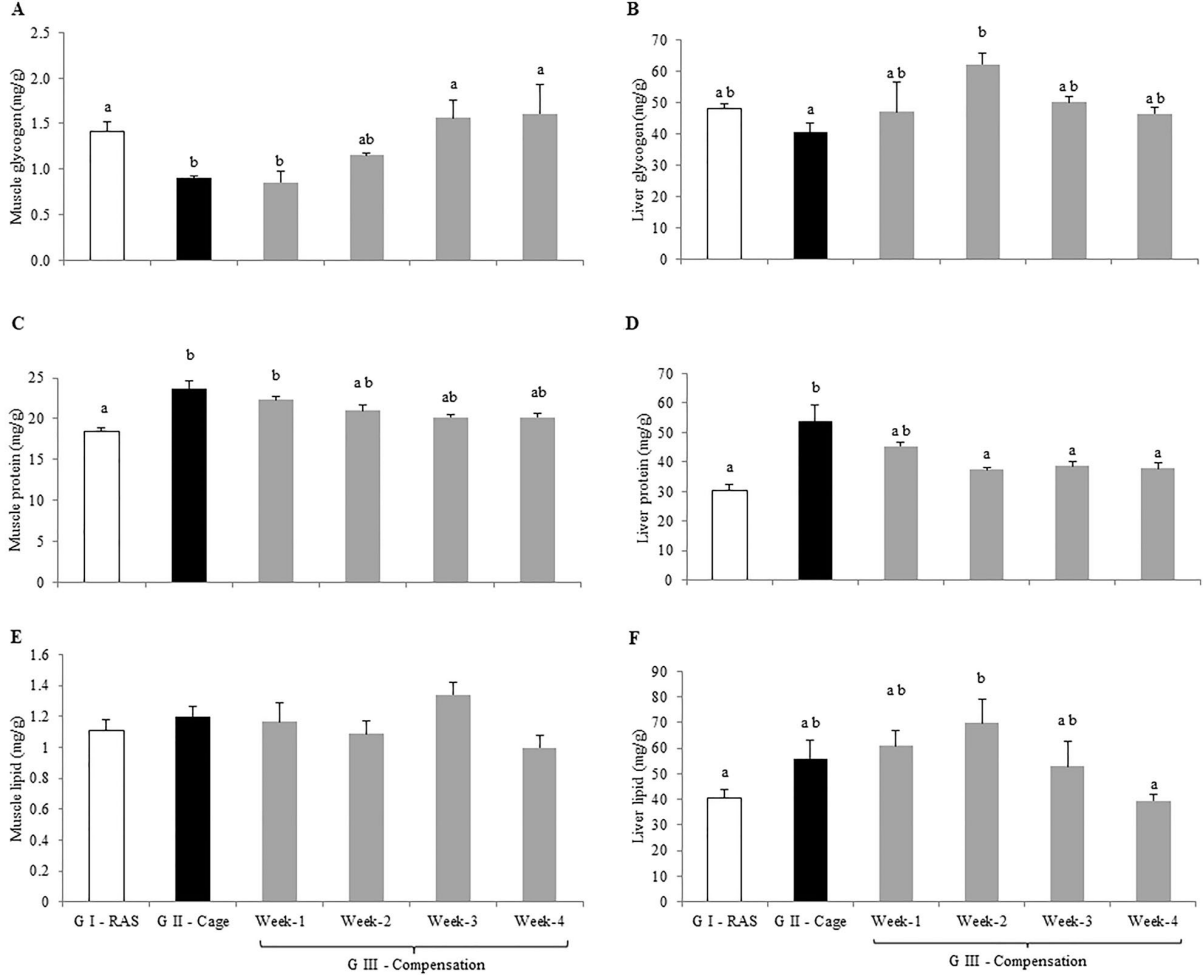
Total energy of (**A**) muscle glycogen (**B**) liver glycogen (**C**) muscle protein (**D**) liver protein (**E**) muscle lipid and (**F**) liver lipid levels of hybrid red tilapia *Oreochromis* sp. from Treatment I—RAS (white bar), Treatment II—Cage (black bar) and Treatment III—Compensation (grey bars) for week-1, week-2, week-3 and week-4. All values are means ± standard error of the mean (SEM) (n = 10). Superscript small letters indicates significant differences amongst cultured in different treatments (*p* < 0.05). Since there were no significant differences among the treatments, grouping letters were omitted in Fig. 1E.

Muscle glycogen for Treatment I was recorded at 1.41 ± 0.20 mg/g, which was significantly higher compared to Treatment II at 0.90 ± 0.14 mg/g (*p* < 0.05). Nevertheless in Treatment III, tilapia that adapted to RAS at week-1 of recovery had the lowest muscle glycogen level at 0.85 ± 0.12 mg/g. However, muscle glycogen levels showed a significant increment at week-3 with 1.56 ± 0.20 mg/g and week-4 with 1.61 ± 0.33 mg/g as compared to week-1 (*p* < 0.05; Fig. 1A). While for liver glycogen, the lowest value was recorded in Treatment II at 40.44 ± 2.89 mg/g and the highest liver glycogen level was recorded in Treatment III at week-2 at 62.17 ± 3.59 mg/g (*p* < 0.05; Fig. 1B).

Both muscle and liver protein from Treatment I were recorded at 18.45 ± 0.39 mg/g and 30.31 ± 2.14 mg/g (Fig. 1) respectively, which were significantly lower compared to Treatment II with muscle protein recorded at 23.71 ± 0.87 mg/g and liver protein at 53.80 ± 5.39 mg/g (*p* < 0.05; Fig. 1C). Liver protein content in Treatment II was considerably the highest as compared to Treatment I and Treatment III (*p* < 0.05; Fig. 1D).

**Figure 2:**
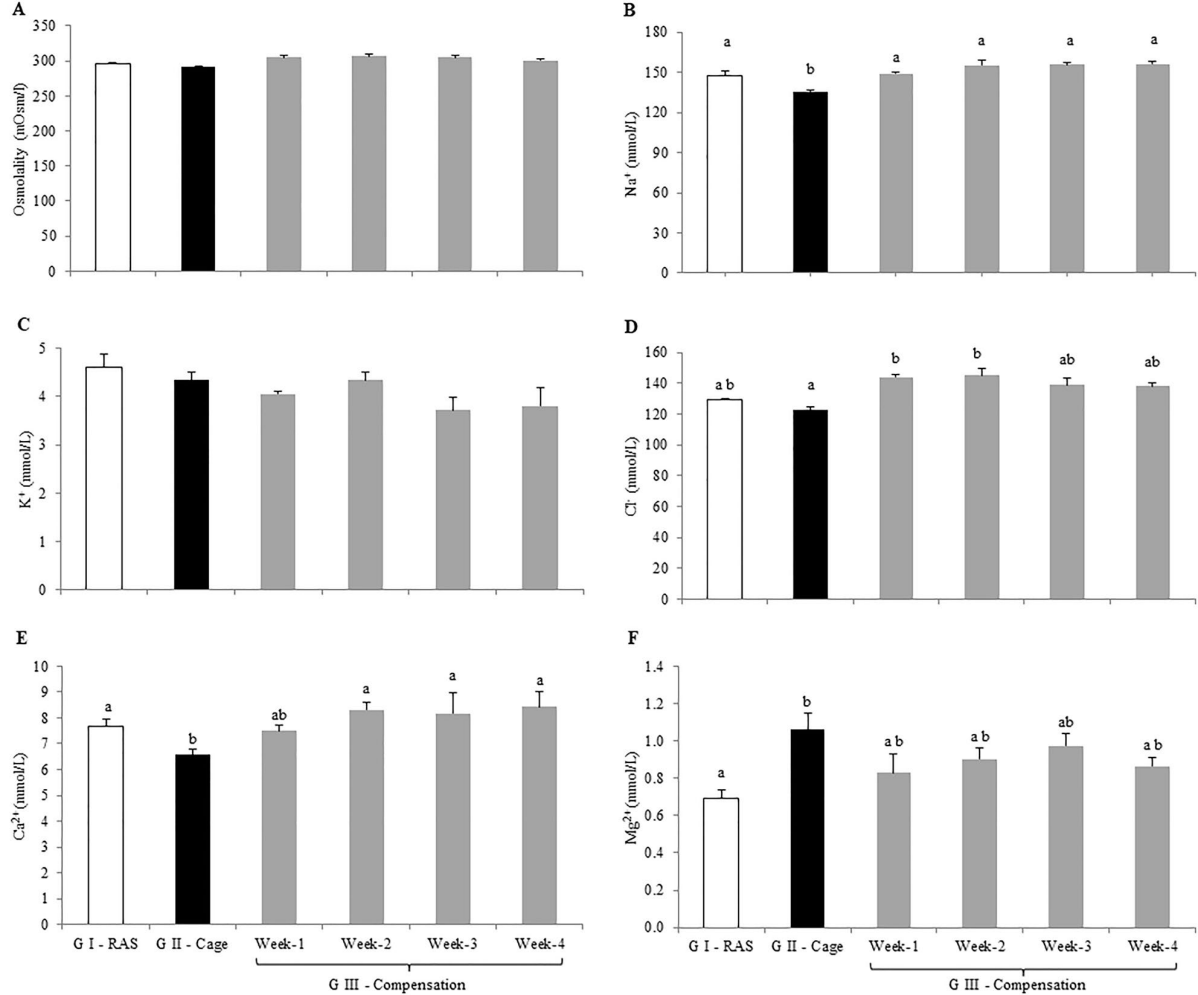
Plasma composition (**A**) osmolality (**B**) Na^+^ (**C**) K^+^ (**D**) Cl^−^ (**E**) Ca^2+^ and (**F**) Mg^2+^ levels of hybrid red tilapia *Oreochromis* sp. from Treatment I—RAS (white bar), Treatment II—Cage (black bar) and Treatment III—Compensation (grey bars) for week-1, week-2, week-3 and week-4. All values are means ± standard error of the mean (SEM) (n = 10). Superscript small letters indicates significant differences amongst cultured fish in different treatments (*p* < 0.05). Since there were no significant differences among the treatments, grouping letters were omitted in Fig. 2A & C.

Muscle lipid levels remained relatively stable among all Treatments of tilapia from Treatment I, II and III (*P* > 0.05; Fig. 1E). Contrastingly, the lowest liver lipid was recorded in Treatment I and Treatment III at week-4 at 40.59 ± 3.44 mg/g and 39.54 ± 2.44 mg/g, respectively (*p* < 0.05; Fig. 2B). Liver lipid level from Treatment II was not significantly different compared to Treatment III at week-1,-2 and -3 (*p* > 0.05; Fig. 1F).

### Plasma osmolality and electrolytes

Plasma osmolality and electrolytes concentration was presented in Fig. 2. Plasma osmolality for all treatments remained relatively stable with 295.54 ± 1.44 mOsm/L recorded for Treatment I and 291.52 ± 1.65 mOsm/L (*p* > 0.05; Fig. 2A). Meanwhile, plasma osmolality level in Treatment III was recorded at a range of 300.05 ± 1.78 mOsm/L to 306.28 ± 2.81 mOsm/L, which were relatively stable within 4 weeks (*p* > 0.05; Fig. 2A). The lowest plasma sodium (Na^+^) was found from Treatment II with only 135.39 ± 1.60 mmol/L compared to all other treatments (*p* < 0.05; Fig. 2B). Plasma Na^+^ levels for Treatment I and Treatment III for all weeks were maintained relatively stable at a range of 146.95 ± 4.23 mmol/L to 156.24 ± 7.69 mmol/L (*p* > 0.05; Fig. 2B). Potassium (K^+^) is the second important cation for biological processes in organism. This study found that plasma K^+^ levels were stable in all treatments ranging from 3.71 ± 0.78 to 4.60 ± 0.89 mmol/L (*p* > 0.05; Fig. 2C). Differently for plasma chloride (Cl^−^) levels, the lowest plasma Cl^−^ was noted from Treatment II at 122.58 ± 1.82 mmol/L, while the highest plasma Cl^−^ were detected in Treatment III at week-1 and week-2 at 143.85 ± 1.48 mmol/L and 145.05 ± 4.56 mmol/L, respectively (*p* < 0.05; Fig. 2D). Similar trend was also noticed for plasma calcium (Ca^2+^) with the lowest value observed from Treatment II at only 6.57 ± 0.29 mmol/L compared to other treatments (*p* < 0.05; Fig. 2E). Plasma Ca^2+^ from Treatment I and III remained insignificantly different ranging from 7.58 ± 0.26 mmol/L to 8.43 ± 0.59 mmol/L (*p* > 0.05; Fig. 2E). Whereas for plasma magnesium (Mg^2+^), lowest value was noticed in tilapia from Treatment I with only at 0.69 ± 0.04 mmol/L (*p* < 0.05; Fig. 2F). Overall, highest plasma Mg^2+^ was noticed in tilapia from Treatment II at 1.05 ± 0.09 mmol/L, but was not significantly different compared to Treatment III ranging from 0.82 ± 0.12 mmol/L to 0.97 ± 0.07 mmol/L (*p* > 0.05; Fig. 2F).

### Gills and kidney enzymatic Na^+^/K^+^ ATPase activity

**Figure 3:**
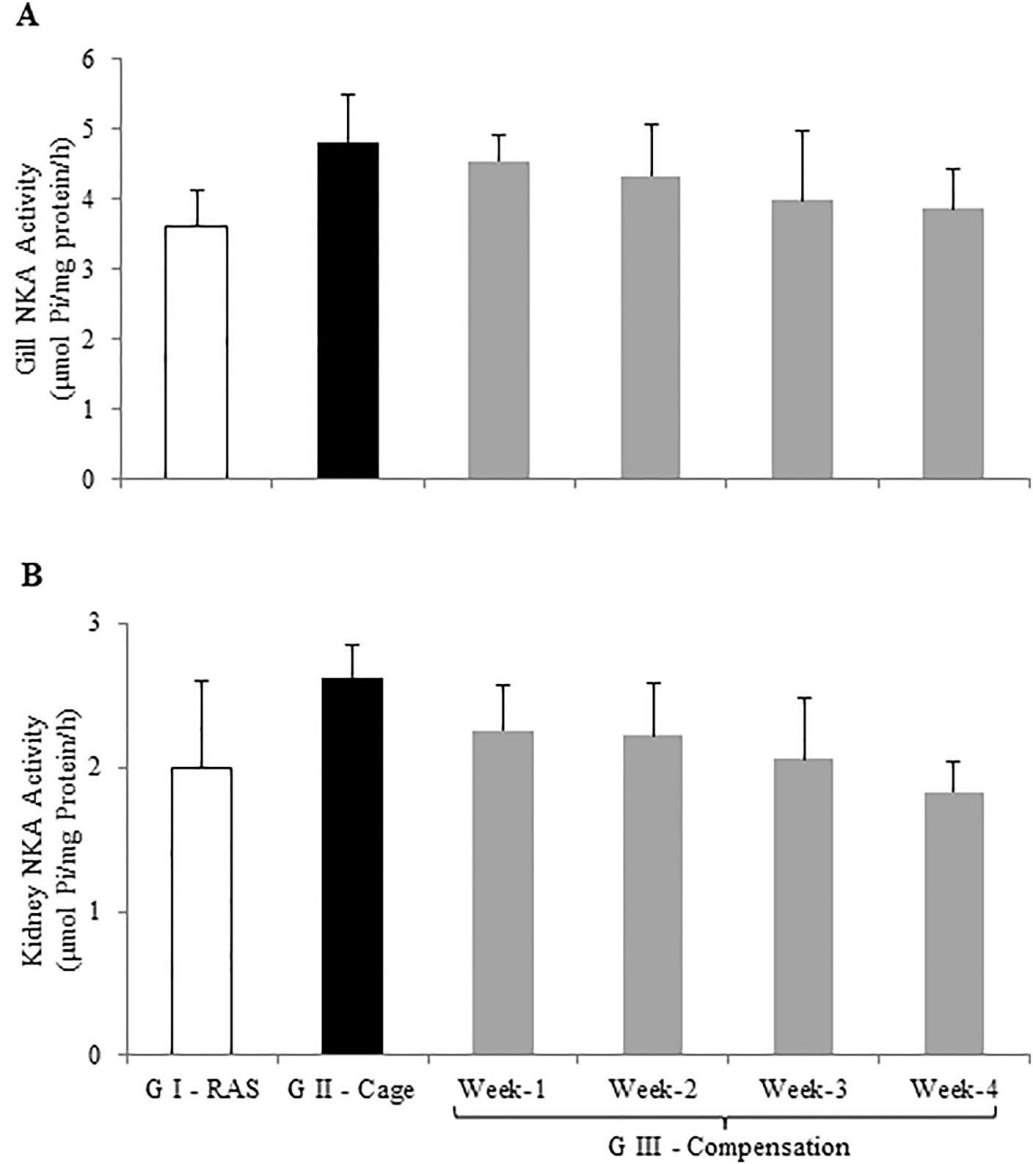
(**A**) Gills and (**B**) kidney Na^+^/K^+^ ATPase (NKA) activities levels of hybrid red tilapia *Oreochromis* sp. from Treatment I—RAS (white bar), Treatment II—Cage (black bar) and Treatment III—Compensation (grey bars) for week-1, week-2, week-3 and week-4. All values are means ± standard error of the mean (SEM) (n = 10). Superscript small letters indicates significant differences amongst cultured in different treatments (*p* < 0.05). Since there were no significant differences among the treatments, grouping letters were omitted in Fig. 3A & B.

Sodium pump or NKA for the gills and kidney of tilapia cultured under different environment were presented in Fig. 3. In general, both gills and kidney NKA activities were expressed in the similar pattern (Fig. 3A and B). There were no differences in gill (Fig. 3A) and kidney (Fig. 3B) NKA activities of tilapia cultured under different environments (*P* > 0.05).

## Discussion

### Growth and energy mobilisation pattern of tilapia living in different environments

Hepatosomatic index (HSI) is defined as the ratio of liver mass to body weight, where HSI is commonly used as a reference to define the status of feeding and nutrition intake^18^ of an organism with energy storage for growth and reproduction^19^. As data obtained in this study, lower HSI was observed in Treatment II which indicate the prioritisation of energy to support high exercise activity and metabolic rates. Where tilapia from Treatment II were noticed to swim actively in a circle from the observation, therefore we speculated that this swimming behaviour might led to high energy expenditure for exercise while maintaining efficient aerobic metabolism. Thereby, active swimming accelerates energy mobilisation, thus reduced HSI. In contrast, tilapia in Treatment I and Treatment III have relatively higher HSI values compared to Treatment II. This is because the tilapia from Treatment I and III cultured in the RAS system had low exercise capacity and suitable condition for mating, thus reserves energy for secondary maturation and reproduction. Evidently, this was noticed in Treatment III tilapia with significant HSI increment till week-3 and started to reduce on week-4, which was believed to associate with energy mobilisation for reproduction purpose. Although, fecundity data is not reported as no eggs was collected, however, during the experimental observation noticed that male fishes started territorial defending behaviour. Previous study also referred HSI as a good indicator of total energy reserves on Atlantic cod *Gadus morhua*^20^.

As the HSI reflected the total energy reserved in the liver of tilapia, this is obviously perceived that the tilapia from Treatment I mobilised both liver protein and lipid to support the reproduction process while reserving glycogen for routine metabolism needs. This was supported with the spawning process and mouth incubation occurred during the study period. Similar energy mobilisation pattern was noticed in Treatment III showing a significant energy reserved for the first two weeks and then mobilised liver protein and lipid on week-3 onwards to support gonadogenesis. At week-4, liver protein and lipid reached to the similar level as tilapia from Treatment I with mouthbrooding behaviour noticed in this study.

Both HSI and energy mobilisation pattern support GSI development as an indication of readiness for spawning process especially for tilapia from Treatment III with an increasing GSI. This is also in agreement with our hypothesis that RAS condition (Treatment I and Treatment III) was more suitable for spawning as the bottom of the tank provided spawning grounds and territorial site to allow collection of fertilized eggs by female tilapia, hereby, reducing somatogenesis and growth performance. As compared to Treatment II, tilapia lived in condition without the bottom base which was difficult for fertilization and eggs collection processes. Therefore, tilapia in this treatment tend to prioritise their energy for somatogenesis, thus low GSI was obtained in tilapia from Treatment II. This phenomenon also reported previously proven that cage-cultured tilapia tends to have very low fecundity or no spawning observed depended on cage mesh size due to difficulties in eggs collection^2,21^.

Glycogen is one of the important energy sources to maintain basal metabolism^22^ and serves as a readily energy supply to meet metabolic needs under unfavourable environmental challenges^22^. This corresponded to the tilapia from Treatment II having the lowest liver glycogen, which was believed to be related to their exercise capacity in the cage. On the other hand, muscle glycogen reached the lowest level on week-2 and was restored to a higher level on week-3 onwards from Treatment III possibly related to the acclimation process from cage (Treatment II) to RAS (Treatment III) conditions in the first two weeks. Meanwhile, liver glycogen peaked at week-2 and returned to a similar level as tilapia from Treatment I and Treatment III at week-1, -3 and -4, although the liver glycogen levels were not significantly different. This showed that tilapia reserved liver glycogen as readily energy to support any spontaneous activity when required.

In fish, protein is known to be more important for somatogensis and efficiently catabolised into energy sources to support aerobic metabolism as compared to lipids and glycogen^6,23,24^. This is in agreement with the results obtained in this study. The Treatment II tilapia cultured in floating cage did not spawn, therefore protein was reserved for somatogenesis as higher muscle and liver protein were recorded. Whereas, protein level for tilapia from Treatment I and III were displayed at a relatively similar level which were lower compared to Treatment II. Low level of protein content was believed to being mobilised to support the mating and spawning processes^4^. This was in agreement with previous studies reported that at mature age, fish tend to reserve protein as energy source and use to support gonadosomatic development^25–27^. Protein is also known as a central role in production that allows the fish to reallocate energy used for growth to other metabolic needs at different life stages based on priority^9,28,29^ as well as to improve adaptability performance in different environmental changes^30^.

On the other hand, lipid mobilisation was not notified in the muscle, but liver lipid mobilisation was distinguished in tilapia from Treatment I and Treatment II week-4. Significant liver lipid mobilisation is believed not only to support basal metabolism and somatogenesis, but also to support secondary maturation for reproduction^30^. Energy requirements for gonad maturation appeared to come from liver reserves and it is noted that 1 g of lipid contains 2 times higher energy than 1 g of carbohydrates or 1 g of protein^31–33^.

### Ionoregulation of tilapia living in different environments

Higher plasma osmolality was recorded in Treatment II tilapia indicated that tilapia increased their osmolality to facilitate active ion uptake as well as enhance metabolites such as glucose and/or glycogen mobilisation to support routine and active metabolic activities. Facilitating active ion uptake was in parallel with high NKA activity found in both gills and kidney. Accordind to Morgan et al.^31^, maintaining or increasing plasma osmolality is important to conserve stable ionic concentration in body fluid with support of active osmoregulation. High NKA activities in tilapia from Treatment II were also believed to associate with swimming activity as highlighted in goldfish and common carp when forced to swim, significantly accelerated the gills NKA actively^5,7^. Contradictory, lower plasma Na^+^ was noticed from Treatment II tilapia, while plasma Na^+^ in tilapia from Treatment I and III were relatively stable. Although higher NKA activities in gills and kidney were noticed from Treatment II, this does not retain Na^+^ level efficiently. Loss of Na^+^ might occur from Treatment II tilapia could be associated with the living condition in the lake water which relatively contained low ionic levels compared to tilapia that lives in the RAS water (Table 1). In Treatment III, gills and kidney NKA activities were relatively stable similar to the tilapia from Treatment I, where both tilapia were living under similar conditions.

On the other hand, plasma K^+^ levels were relatively stable, except in tilapia from Treatment I that had a slightly higher K^+^ level. High plasma K^+^ is believed to be released from the tissue into the bloodstream in cooperating NKA activity not only to facilitate the Na^+^ uptake but also helped in ammonia excretion^7^. Another explanation was associated with the defensive behaviour of tilapia from Treatment I, due to active defensive territorial activities for mating that resulted in high metabolic rate. Active exercise resulted in tissue K^+^ leaked into the body fluid was reported in common carp^32^. Na^+^, K^+^ and Cl^−^ are important ions that provide the sustainable osmotic pressure of body fluids to regulate homeostasis and acid–base balance^33,34^. As essential ion, plasma Cl^−^ level was maintained consistently in tilapia from Treatment III, but slightly decreased in tilapia from Treatment I and II. The stability of plasma Cl^-^ was probably correlated with an increase in Na^+^ uptake via the Na^+^/Cl^−^ exchanger through dietary intake^5^. Feeding is known to provide an excessive base which consequently led to the uptake of Cl^−^ via branchial Cl^-^/HCO_3_ exchanger as reported in rainbow trout^35,36^. As important ions, the association of unidirectional influx and efflux of Na^+^ and Cl^−^^37^ to be adjusted to a net flux via Cl^−^ uptake at gills Cl^−^/HCO_3_^−^ exchanger was noted to avoid alkalosis metabolism^38^.

As all fishes in all treatments were fed twice daily, dietary Ca^2+^ uptake seem sufficient to support basal metabolic needs. Ca^2+^ is known to play important role in bone and scale formation. Therefore, it is highly essential for tilapia from Treatment II and III to maintain sufficient level of Ca^2+^ to support basal metabolic needs. Higher plasma Ca^2+^ levels shown a strategy to retain Ca^2+^ via active uptake through Ca^2+^ transporter and Ca^2+^ channel. In addition, an increasing trend of Ca^2+^ in Treatment I and III were believed that the tilapia having high hepatic vitellogenin production invest for reproduction, which had been proven previously that vitellogenin carrier of important ions including calcium for egg yolk development in teleost^39^. Mg^2+^ is the second most abundant cation exists in the intracellular fluid that acts as a functional co-factor for enzymes as well as plays an important role in neurochemical impulse transmission and muscle excitability^34^. Changes in plasma Mg^2+^ level are always associated with stress or environmental changes^40,41^. This phenomenon was noticed in this study where plasma Mg^2+^ levels were inconsistent, where higher Mg^2+^ level was found in tilapia from Treatment II, lower in tilapia from Treatment I and fluctuated in tilapia from Treatment III. It was believed that the different levels of ions were influenced by the living conditions in association with territorial competition for mating and water ionic status, especially Treatment II condition.

## Conclusion

The present study revealed that tilapia mobilized their energy differently under different cultured environment. Tilapia preference in accessing glycogen as an easy energy to support exercise metabolism under cage cultured condition, while tilapia cultured in RAS mobilised lipid and protein for gonadagenesis purposes. The gills and kidney NKA activities in all treatments of tilapia remained steady to support balance homeostasis for basal metabolism, without being influenced by living conditions.

## Data Availability

All the data presented in this study are provided within the main manuscript. The datasets generated during and/or analysed during the current study are available from the corresponding author on reasonable request.
